# Applications and challenges of CRISPR-Cas gene-editing to disease treatment in clinics

**DOI:** 10.1093/pcmedi/pbab014

**Published:** 2021-07-10

**Authors:** Wenyi Liu, Luoxi Li, Jianxin Jiang, Min Wu, Ping Lin

**Affiliations:** Wound Trauma Medical Center, State Key Laboratory of Trauma, Burns and Combined Injury, Daping Hospital, Army Medical University, Chongqing 400042, China; Wound Trauma Medical Center, State Key Laboratory of Trauma, Burns and Combined Injury, Daping Hospital, Army Medical University, Chongqing 400042, China; Wound Trauma Medical Center, State Key Laboratory of Trauma, Burns and Combined Injury, Daping Hospital, Army Medical University, Chongqing 400042, China; Department of Biomedical Sciences, School of Medicine and Health Sciences, University of North Dakota, Grand Forks, North Dakota 58202–9037, USA; Wound Trauma Medical Center, State Key Laboratory of Trauma, Burns and Combined Injury, Daping Hospital, Army Medical University, Chongqing 400042, China; Biological Science Research Center, Southwest University, Chongqing 400716, China

**Keywords:** gene editing, clinical trials, hereditary diseases, cancer, gene therapy, viral vectors

## Abstract

Clustered regularly interspaced short palindromic repeats (CRISPR)-associated systems (Cas) are efficient tools for targeting specific genes for laboratory research, agricultural engineering, biotechnology, and human disease treatment. Cas9, by far the most extensively used gene-editing nuclease, has shown great promise for the treatment of hereditary diseases, viral infection, cancers, and so on. Recent reports have revealed that some other types of CRISPR-Cas systems may also have surprising potential to join the fray as gene-editing tools for various applications. Despite the rapid progress in basic research and clinical tests, some underlying problems present continuous, significant challenges, such as editing efficiency, relative difficulty in delivery, off-target effects, immunogenicity, etc. This article summarizes the applications of CRISPR-Cas from bench to bedside and highlights the current obstacles that may limit the usage of CRISPR-Cas systems as gene-editing toolkits in precision medicine and offer some viewpoints that may help to tackle these challenges and facilitate technical development. CRISPR-Cas systems, as a powerful gene-editing approach, will offer great hopes in clinical treatments for many individuals with currently incurable diseases.

## Introduction

The advent of clustered regularly interspaced short palindromic repeats (CRISPR)-associated systems (Cas) systems has revolutionized the gene-editing field for research, biotechnology, and, potentially, disease treatment in clinics. This technology possesses excellent features for manipulating genomes, such as easy design, low costs, rapid turnaround time, and particularly its high accuracy and efficiency. Hence, CRISPR-Cas systems have multiple advantages and have overtaken the earlier-used gene-editing tools [e.g. zinc-finger nucleases (ZFNs), transcription activator-like effector nucleases (TALENs)].^1^ In the past few years, researchers have utilized CRISPR-Cas tools to edit genomes in almost all organisms including human cells, primates, mice, zebrafish, *Bombyx mori*, and tiny microorganisms.^2–4^ These endeavors indicate that the CRISPR-Cas system has opened a new avenue for preclinical and clinical research for treating a spectrum of refractory diseases. A number of monogenic human genetic diseases afflict human beings, and there have been several preclinical/clinical trials using the CRISPR-Cas system to reverse the underlying genetic causes to treat these diseases.^5^ Additionally, some diseases with multiple or complicated genetic mutations may be ameliorated by the correction of genetic codes with CRISPR-Cas. Furthermore, the CRISPR-Cas system has been developed to fulfill its potential as a feasible treatment option for some infectious diseases, autoimmune disorders, and cancers.^6,7^ In 2018, the first *ex vivo* clinical trial using cells edited with Cas9 was approved for treating cancer.^8^ In 2019, the first *in vivo* clinical trial was approved by the Food and Drug Administration.^9^ Thereafter, a growing number of clinical trials are currently underway (https://clinicaltrials.gov). Despite the promise for therapeutic applications, there are a range of challenges going forward that should be resolved before the realization of clinical treatments in patients with different diseases using CRISPR-Cas technologies.

## Types of CRISPR-Cas systems

**Figure 1.**
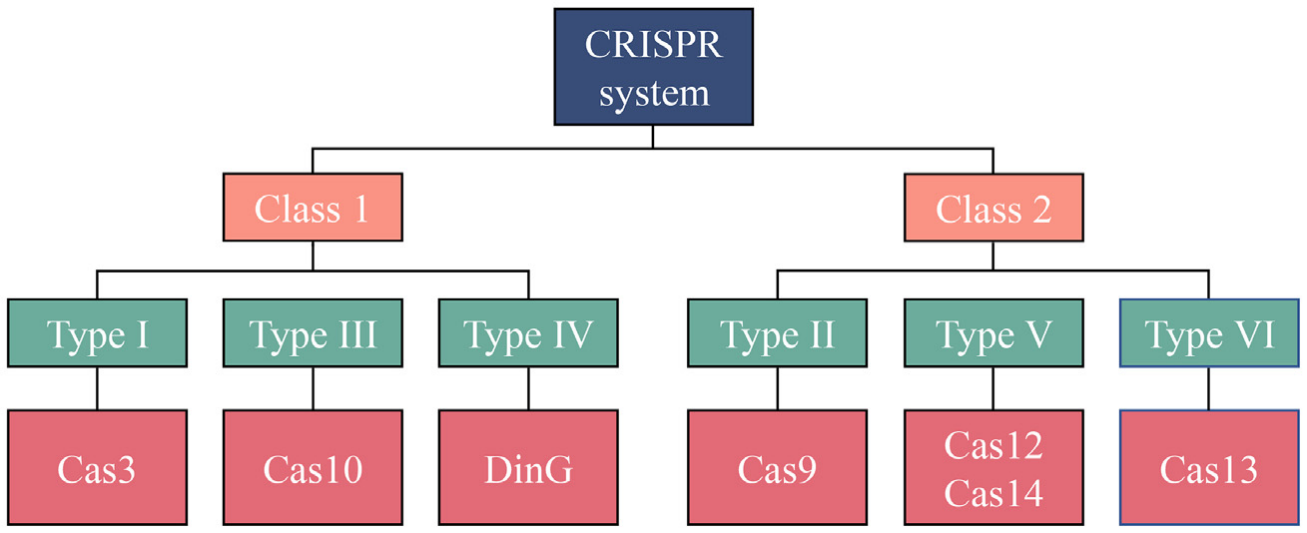
A schematic representation of the CRISPR system and associated nucleases. The CRISPR-Cas systems have been categorized into two classes (I and II) and subdivided into six types (I–VI) with diverse subtypes. The class 1 CRISPR-Cas system includes types I, III, and IV, employing representative endonucleases of Cas3, Cas10, and DinG, respectively. The class 2 CRISPR-Cas system includes types II, V, and VI, to cleave RNA-guided genetic codes with Cas9, Cas12–Cas14, and Cas13, respectively.

Due to differences of core Cas proteins, CRISPR-Cas systems have been categorized into two classes (1 and 2) and subdivided into six types (I–VI) with diverse subtypes. The class 1 CRISPR-Cas system, which functions through a multi-Cas protein complex, includes types I, III, and IV, employing representative endonucleases of Cas3, Cas10, and DinG, respectively. The class 2 CRISPR-Cas system, which employs single Cas protein, includes types II, V, and VI, to cleave RNA-guided genetic codes with Cas9, Cas12–Cas14, and Cas13, respectively (Fig. 1). Types I, II, and V systems are shown to specifically target DNA, and type III targets both RNA and DNA; type VI can only edit RNA. The function and mechanisms of type IV system remain largely unknown.^10,11^

With the aid of guide RNA, the CRISPR-Cas system is capable of performing gene insertion (knockin novel genes), deletion (knockout existing genes), and base editing in DNA or RNA. Among them, base editing is a newer gene-editing method for generating precise point mutations in DNA or RNA by utilizing components of CRISPR systems together with some other enzymes.^12^ There are two DNA base editors: adenine base editors (ABEs) and cytosine base editors (CBEs). The ABEs convert the A–T pair into a G–C base pair, while CBEs help the transition of the C–G base pair to a T–A pair.^13,14^ Although all four types of nucleotides can be edited by these two enzymes, a major limitation for base editing is that the surrounding region may contain multiple “bystander” cytidines or adenines. Furthermore, C or A targeting only occurs within 15 nucleotides (nt) of a protospacer adjacent motif (PAM) sequence. RNA base-editing systems have been divided into two classes, the RNA Editing for Specific C-to-U Exchange (RESCUE) system induces C-to-U replacement, and the RNA Editing for Programmable A to I Replacement (REPAIR) system exerts A-to-I (G) replacement.^15,16^

Cas9 is the first and most in-depth researched nuclease and has been widely used to provide a simple and affordable way to mediate the manipulation and editing of DNA. Cas9 utilizes the pattern of complementary base pairing to recognize and edit the sequences of target DNAs with the aid of guide RNA. The requirement of a short DNA sequence (PAM) for editing DNAs may limit Cas9’s potential in selecting target sites.^17^ Moreover, DNA editing may target a gene in an operon, which could result in the side effect of silencing the transcription of downstream genes.^18^ These issues need to be considered when using the CRISPR-Cas9 system in gene editing. With the hope of developing tools with lower off-target effects than RNAi for modulating gene expression at the translation stage, the system of Cas13 has been developed into a versatile RNA base-editing tool. The CRISPR-Cas13 system has rapidly gained great attention. To date, researchers have identified six Cas13 protein families: Cas13a (C2c2),^19^ Cas13b,^20^ Cas13c,^21^ Cas13d,^22^ Cas13X,^23^ and Cas13Y.^23^ Cas13X and Cas13Y have been the most recent discoveries, which are as small as 775–803 amino acids, making their application much easier and versatile. The engineered Cas13X.1 (775 aa), which could tolerate single-nucleotide mismatches in recognition, can interfere with RNAs in mammalian cell lines. Truncated Cas13X.1 (445 aa) and engineered deaminase (385 aa) comprise a minimal RNA base editor, which shows high specificity and robust editing efficiency to lead RNA base conversions.^23^ The diversity and evolution of the CRISPR-Cas systems provide the opportunity to expand toolboxes for researchers and clinicians to improve and refine genome editing for practical use.

## Applications of CRISPR-Cas systems in COVID-19

The coronavirus disease 2019 (COVID-19), which is caused by severe acute respiratory syndrome coronavirus-2 (SARS-CoV-2) and reported for the first time in late 2019, is the most deadly pandemic in our lifetime, affecting more than 215 countries or areas all over the world.^24^

The CRISPR-Cas systems have been developed to be quick and specific methods for SARS-CoV-2 diagnosis.^25–27^ Compared to the routine molecular diagnostic method reverse transcription-quantitative polymerase chain reaction (RT-qPCR), CRISPR-based SARS-CoV-2 diagnostic detection may be cheaper, more specific, more sensitive, and has no need for complex instruments. Hence, this novel approach may have the potential for accelerated detection and convenient use at the point-of-care. Meanwhile, CRISPR-facilitated detection may produce more reliable tests by eliminating or reducing false negative, false positive, or uncertain results of RT-qPCR.^28^

In addition, CRISPR-Cas systems have also been tested for therapeutic application in COVID-19. PAC-MAN (prophylactic antiviral CRISPR in human cells) is such a CRISPR-Cas13-based strategy, which utilizes the RNA-guided RNA endonuclease activity of Cas13d in human cells to eliminate the SARS-CoV-2 virus. Specifically, this study indicated that the CRISPR-Cas13d system can be used to effectively target and cleave the RNA sequences of SARS-CoV-2 fragments with properly designed guide RNAs in lung epithelial cells. Moreover, bioinformatics analyses suggested that as few as 6 crRNAs can target 91% of 3051 sequenced coronaviruses, which may help us to be ready for future pandemics caused by coronaviruses.^29^ PAC-MAN is a proof-of-concept antiviral strategy, and the CRISPR-Cas13 system might be an alternative therapeutic strategy for COVID-19, particularly with improved versions of reliable, safe, and efficient delivery systems.^30^ The therapeutic application of CRISPR-Cas13 systems in treating COVID-19 requires an available *in vivo* delivery method, and PAC-MAN will need to be validated in relevant preclinical models, such as rhesus macaques, to test its antiviral efficacy, specificity, and Cas13d-induced immunogenicity. PAC-MAN may be a potential strategy to be used against COVID-19 and future viral threats.

## Preclinical tests

A growing number of preclinical studies based on rodent and other animal models indicate that CRISPR-Cas systems have the potential for therapeutic usage in different diseases, including genetic diseases,^31^ infectious diseases,^32^ cancers,^7,33^ immunological diseases (autoimmunity and immunodeficiency),^34^ etc.

Monogenic diseases, which are associated with more than 75 000 genetic variants, affect a large population of patients, and sadly the majority of them remain difficult to treat.^35^ The CRISPR-Cas tools are being widely used to correct genetic variants with the hope of treating many human genetic diseases, such as inherited blood disorders (sickle cell disease, β-thalassemia, and hemophilia),^36–38^ inherited eye diseases (Leber congenital amaurosis and inherited retinal degeneration),^39–41^ muscular genetic disease (Duchenne muscular dystrophy),^42–44^ genetic liver diseases (α-1 antitrypsin deficiency and hereditary tyrosinemia type 1),^45–47^ congenital genetic lung disease (inherited surfactant protein syndromes and cystic fibrosis),^48–50^ neurological disorders (Parkinson's disease, Alzheimer's disease, and Huntington's disease),^51–53^ genetic deafness,^54,55^ etc.

In addition to the treatment of monogenic diseases, CRISPR-Cas systems have been utilized to treat infectious diseases, such as viral infections (human immunodeficiency virus infection, hepatitis virus infections, and oncogenic virus infections), nonviral infections (bacterial, fungal, and parasite infections), and the potential efficacy has been observed in a number of occasions.^56^ Acquired immunodeficiency syndrome (AIDS) caused by infection of human immunodeficiency virus (HIV) is still one of the world's most serious public health challenges.^57^ Yin et al. reported that the CRISPR-Cas9 tool could inhibit multiple steps of HIV-1 infection,^58^ and several research labs have made excellent efforts to improve the use of CRISPR-Cas9 for treating HIV infection.^59–61^ Recently, CRISPR-Cas12 and CRISPR-Cas13 systems have been used to inhibit HIV infection.^62,63^

A variety of studies have applied CRISPR-Cas systems for effectively targeting different genes and have managed to prove the potential treatment ability for initiation or progression of lung cancer,^64^ breast cancer,^65,66^ and many other types of cancers.^67–69^ Meanwhile, the CRISPR-Cas system has been harnessed to serve as a powerful tool with the ability of unbiased screening of precision medicine including identification of new drug targets, biomarkers, and elucidation of mechanisms leading to drug resistance.^70–72^ In short, there are tremendous potential applications for CRISPR-Cas and their derivative systems (i.e. dCas9) due to the ability to accurately determine the underlying disease causes, genetic mutation variants, immunological regulatory factors, cell signaling mediators, and drug targets as well as drug molecules and therapeutics.

## Clinical tests and applications

**Figure 2.**
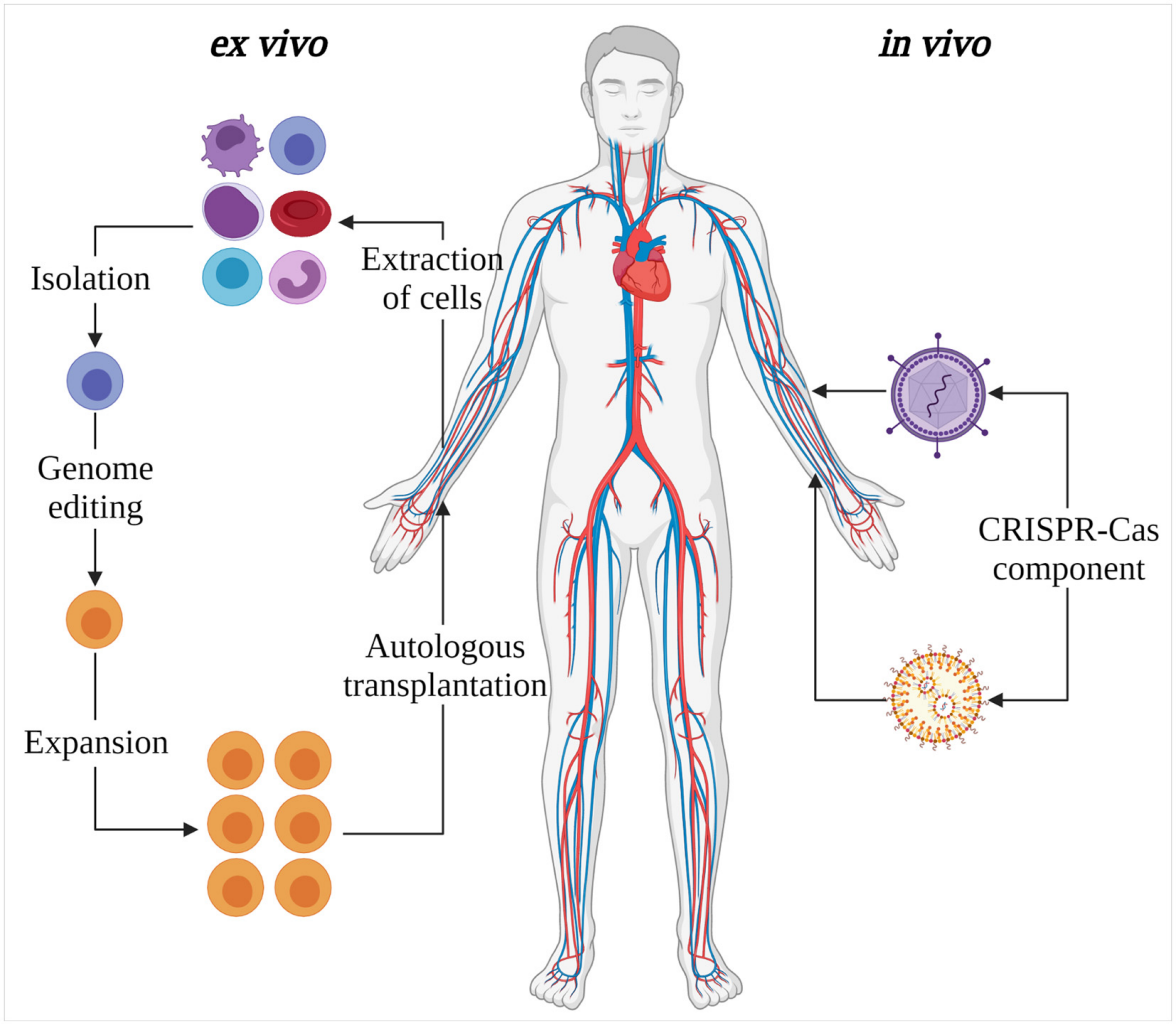
Clinical strategies for CRISPR-Cas system. For *ex vivo* application (left), targeted cells of a patient are extracted, isolated, edited, expanded, and delivered back to the same patient. For *in vivo* application (right), the CRISPR-Cas system is delivered by various vectors to disease-associated cells or organs of the body to correct the mutations or treat the cause of diseases. The figure was created using BioRender.com.

Clinical tests or applications of CRISPR-Cas systems could be categorized into these two classes, *ex vivo* and *in vivo* therapeutic usages (Fig. 2).

For *ex vivo* applications, the cells of a patient are isolated, manually edited, and delivered back to the same patient. The *ex vivo* gene editing method has three main potential applications in clinics: cancer immunotherapy,^73^ treatment of hereditary diseases (e.g. sickle cell anemia, β-thalassemia, etc.),^74,75^ and viral infection inhibition.^76^

The most clinically advanced application using the CRISPR-Cas9 system focuses on cancer immunotherapy. The first *ex vivo* clinical experiment was carried out in West China hospital of China to treat metastatic nonsmall-cell lung cancer (ClinicalTrials.gov: NCT02793856).^8^ In this study, the peripheral blood of patients was drawn out, and electroporation of Cas9 plus sgRNA plasmid was used to target the PD-1 gene in T cells of the blood. After these processes, it was injected back into the same patients. Of late, the same group led by Dr. You Lu at West China Hospital in China reported for evaluating the feasibility and safety of this CRISPR-Cas9 gene-editing strategy for clinical use.^77^ Despite showing some therapeutic benefits, the current trials may have limited therapeutic efficacy and uncertain safety that needs improvement with further testing.

As examples of hereditary diseases, sickle cell anemia and β-thalassemia were examined in clinical trials for treatment effects by disrupting the erythroid enhancer to the *BCL11A* gene (ClinicalTrials.gov: NCT03655678, NCT03745287). In these studies, patients' hematopoietic stem cells (HSCs) were extracted from their peripheral blood, expanded *ex vivo*, and treated with Cas9/sgRNA against *BCL11A*, a negative regulator of fetal hemoglobin (HbF). Suppressing *BCL11A* can change the dormant state of HbF in adults to compensate for the hemoglobin, and the total hemoglobin levels of two β-thalassemia patients elevated to normal levels 9 months following treatment. While longer follow-up on treatment effects and safety needs to be conducted, these developments may be promising for treating these refractory genetic diseases.

Another *ex vivo* application of CRISPR-Cas system is to inhibit viral replication and spread to treat AIDS. Xu et al. have established a CRISPR/Cas9-mediated CCR5 (the main co-receptor of HIV) ablating system in long-term HSCs, which was purposed to inhibit HIV infection in mice.^78^ In a recent clinical trial (ClinicalTrials.gov: NCT03164135), allogenic CCR5-edited HSCs were transplanted to treat a single patient with HIV and related acute lymphoblastic leukemia. Donor CCR5-depleted cells in the body can be detected after more than 19 months and no major gene-editing-related side effects were observed. This study represents a new method of controlling HIV infection although further research is needed to improve effectiveness.^79^

For *in vivo* applications, the CRISPR-Cas system is delivered to disease-associated organs or cells of the patient to correct the mutations or treat the cause of diseases. Currently, the main application of *in vivo* therapy is to treat monogenic genetic disorders.^80^ In 2019, the first *in vivo* clinical trial was registered (ClinicalTrials.gov: NCT03872479). It is also the first trial where CRISPR-Cas9 systems were directly injected into a human body. Namely, the CRISPR-Cas9 gene therapy-based drug AGN-151587 was directly injected into the subretinal space of patients who suffered from a rare blindness disease named Leber's congenital amaurosis 10, which is caused by gene *CEP290* mutations.^81^ Researchers and doctors are waiting for results about the safety and treatment efficacy.

In addition to these, dozens of registered clinical trials using CRISPR-Cas-mediated gene-editing systems are being carried out all over the world (https://clinicaltrials.gov). However, many underlying obstacles and issues including ethics, safety, and efficacies have emerged and need to be resolved for the development of deliverable CRISPR-Cas applications in clinics. In particular, standardization may be a key, which requires an international governing body to draft a widely acceptable guideline to coordinate clinical trials, assess the outcomes, and make advice/recommendations for future use.

## Editing efficiency

A wide variety of CRISPR-Cas tools have shown their high genome-editing efficiency for a large number of genomic targets; however, going forward, scientists need to try their best to improve their editing efficiency to expand their application range. Multiple factors may influence the editing efficiency of CRISPR-Cas systems, such as double-strand break (DSB) repair mechanisms, guide RNA sequence design, unwanted effects, and delivery efficiency, to name a few.

**Figure 3.**
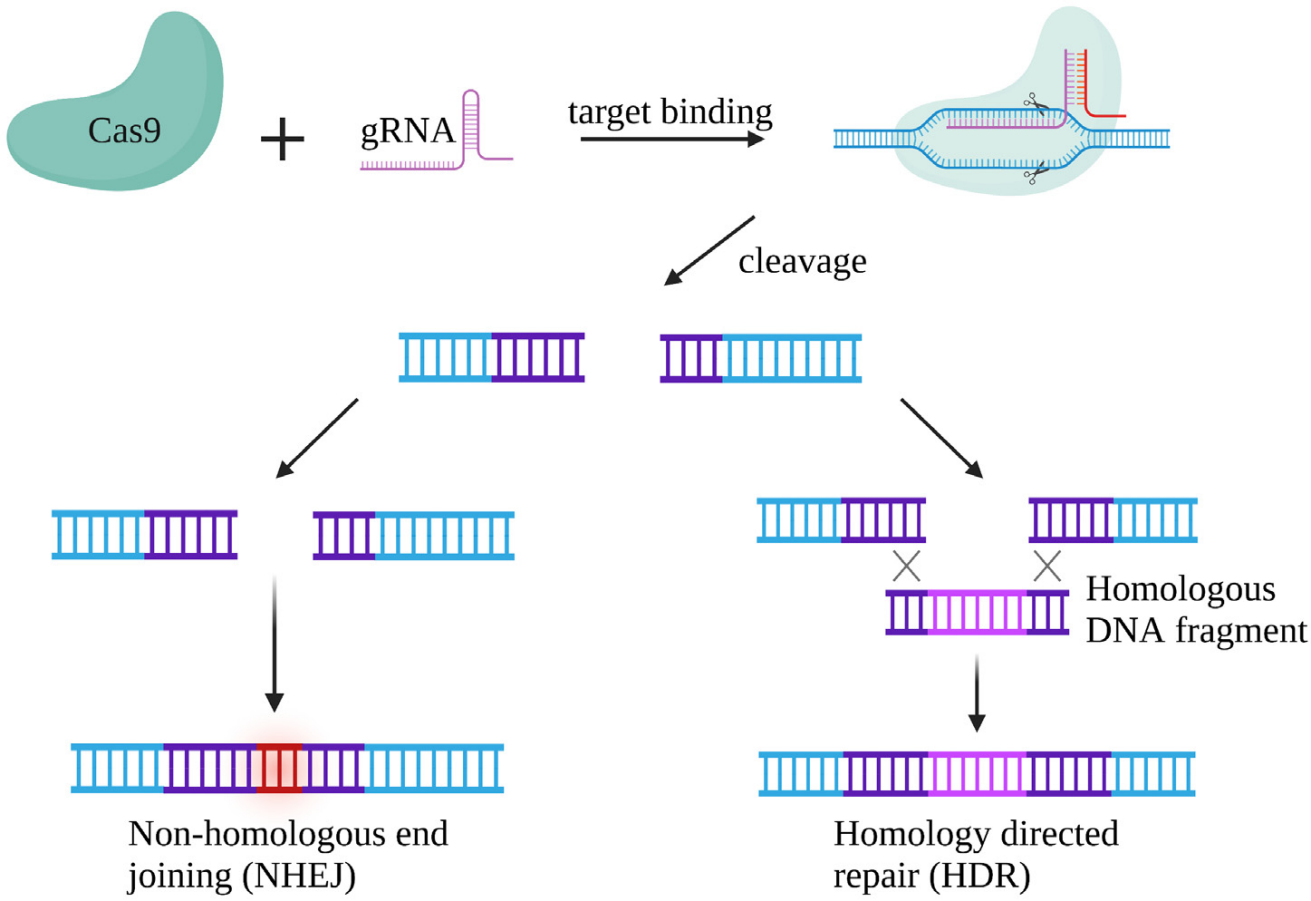
A schematic representation of DSBs repair. DSBs can be repaired by NHEJ or HDR pathways. NHEJ is error-prone as it utilizes no or limited homology, while HR is a precise mechanism using extensive homology. The figure was created using BioRender.com.

A DSB is formed when the CRISPR-Cas system recognizes the target sequences and can be resolved by two mechanisms: homology-directed repair (HDR) to resolve infidelity in editing and nonhomologous end-joining (NHEJ) that potentially gives rise to a large number of errors in eukaryotes and human cells.^82,83^ Accurate modifications and corrections of genetic codes might be compromised by relatively low efficiencies of HDR compared to high frequencies of the NHEJ pathway (Fig. 3). Several methods of suppressing NHEJ mechanisms and enhancing the rates of HDR-mediated repair have been attempted to examine the possibility to improve CRISPR-Cas-mediated gene-editing efficiencies.^84,85^

Furthermore, one of the notable advantages of CRISPR is its simplicity. CRISPR-Cas gene editing relies on only two major components, a gene-targeting guide RNA assisting the Cas protein to induce genetic alterations and the effector Cas endonuclease. The success of CRISPR-associated gene editing depends on the selection of an appropriate guide RNA sequence. To date, several online tools with user-friendly interfaces have been utilized to design and select the optimal guide RNAs with low off-target effects and high editing efficiencies.^86^ Furthermore, even the best-designed guide RNAs may not fulfill the criteria for some applications, which may be engineered by chemical modifications to enhance editing efficiency, improve target specificity, and decrease off-target and biological toxicity.^87,88^ Cas endonucleases are an equally important component of CRISPR activity, which have been developed rapidly but need improvement to help the performance of the CRISPR-Cas system to meet the requirements of many difficult therapeutic applications. Several groups have reported that they have managed to engineer CRISPR-associated nucleases to make CRISPR-Cas systems to be powerful tools for genome editing, and the number of studies just keeps growing.^89–91^ In brief, CRISPR genome editing could be improved by engineering better guide RNAs to target more specifically or by engineering nuclease proteins to edit more effectively.

CRISPR-Cas systems have demonstrated the ability to effectively edit cell lines, but they are often unreliable in editing primary cells, certain tissues, and in patient's bodies. Furthermore, in general, the editing efficiency *in vivo* is much lower than *in vitro*.^87,92^ The reasons for this phenomenon are largely attributed to the lack of efficient delivery methods for Cas endonucleases, which will be discussed in more detail in the next section. Nevertheless, much higher gene-editing efficiencies and fewer nonspecific outcomes will be needed to ameliorate or cure diseases than our currently available CRISPR-Cas toolkits; hence, further research is urgently needed to improve editing efficiencies and reduce the immunogenicity for *in vivo* applications in humans.

## Delivering methods

One of the greatest impediments facing the use of CRISPR-Cas technology in clinics is to attain efficient and specific delivery of CRISPR components. It is a common challenge that every CRISPR-Cas-associated therapeutic needs to deliver a large amount of the genome-editing enzyme into cells, and sometimes delivering multiple macromolecules simultaneously is required.^93^ Scientists have explored various kinds of delivery methods that include three major categories: viral vectors [adeno-associated virus (AAV), adenovirus (AdV), lentivirus (LV), and so on], physical delivery methods (microinjection, electroporation, and hydrodynamic delivery), and nonviral vectors (liposomes and peptide nanoparticles).^94^

The first *in vivo* clinical trial of CRISPR involves the use of AAV vectors.^9^ AAV is thought to not cause any diseases in humans and has a broad range of serotypes that allow for infection of different cells, while not causing an immune response by some specific serotypes. Hence, AAV has become the most widely used delivery vector. However, AAV has a low capacity for packaging genes, which seems a bottleneck for some CRISPR-Cas systems with large molecular sizes.^94–96^ LV and AdV vectors can also be used in CRISPR-Cas therapy systems, and offer better package capacity for delivery compared to AAV. Liu and his team have utilized an LV vector-mediated CRISPR/Cas9 system to knockout HIF-1α to treat human liver cancer. They injected LV vectors containing the Cas9/sgRNA-721 system directly into tumor tissue, and the expression of HIF-1α in tumor cells decreased significantly after treatment.^97^ In a separate study, Koo et al. injected a mixture of AdV expressing Cas9 and AdV expressing EGFR mutation-specific sgRNA into the subcutaneous xenograft nonsmall cell lung cancer model with H1975 cells containing mutant alleles. After three dose treatments, rapid tumor regression was observed and the survival rate was prolonged in the tumor model.^98^ As the diameters of LV and AdV are much larger than that of AAV, they can tolerate larger insertions, which is a significant advantage versus AAV. However, the typical AdVs and LVs might also face significant challenges, such as immune reaction or inflammatory responses in the host.^99,100^ LV tends to integrate into the genome of the host, which may result in persistent gene transfer and the increased propensity of off-target effects. Therefore, when using LV and AdV as vectors, extra caution must be taken.

Physical delivery methods are virus-free and low-cost, but each of them has some disadvantages. Electroporation has predominantly been used *in vitro* to mediate CRISPR-Cas-associated genome editing, and the use of it *in vivo* via direct application of electrode surfaces to some tissues has been reported by some groups.^101,102^ However, using electroporation *in vivo* in the human body is a great challenge, because the electroporation parameters for the tissues of humans are very difficult to choose, and inappropriate parameters may damage the body. Furthermore, microinjection is difficult to operate, and requires injection skills for high accuracy of delivery, and the hydrodynamic delivery approach may be traumatic to tissues.^103,104^ In addition, the deformation of the cell membrane is a potential mode of transmission *in vitro*. The rapid mechanical deformation of the cell membrane may lead to temporary membrane disturbance or holes that may be conducive to the passive diffusion of macromolecules into tumor cells to increase tumor viability. This strategy is suitable for high-throughput transport of almost any macromolecule and has high transfer efficiency. It is reported that the CRISPR/Cas9 system can be transmitted to a variety of tumor cells through a microfluidic device based on membrane deformation.^105,106^

Nonviral vectors have been applied to avoid the safety concerns of viral vectors as they may not incorporate into the human genome to cause permanent mutations.^107^ Among all of the *in vivo* nonviral delivery methods, solid lipid nanoparticle, which is always applied to deliver Cas9 mRNA and gRNA, is the most widely used one.^108,109^ However, the mRNA in solid lipid nanoparticles may induce Toll-like receptor activation, which is harmful to the targeted tissues.^110^ Apart from solid lipid nanoparticles, many other novel nanoparticles have also been investigated for CRISPR-Cas delivery: gold nanoclusters,^111^ gold nanowires,^112^ nanoscale zeolitic imidazole frameworks,^113^ and black phosphorus nanosheets.^114^ These novel nanoparticles are just a bourgeoning area of research. Although many nanoparticles have shown promising results in cell culture, their *in vivo* efficacy and side effects are not very clear, and they often require complex design and it may be difficult to produce high amounts for clinical use.^115^ Furthermore, cationic lipids, such as LipofectAmine, have also been used *in vivo*. Cas9 guided by RNA–lipofectamine complexes has been delivered to the murine ear and successfully ameliorated autosomal dominant hearing loss.^116,117^ Researchers believe that it has the potential for application in other tissues. However, the toxicity of the lipid complex is a major concern that may prevent its clinical application.^93^ In addition, as natural nano-vesicles, exosomes or extracellular vesicles (EVs) that are secreted by epithelial cells, immune cells, and tumor cells can be used to transfer the CRISPR/Cas system with high biocompatibility and low immunogenicity. However, the loading efficiency of exosomes and complex composites needs to be addressed before real clinical application.^118,119^

In sum, every approach has some advantages and disadvantages (Table 1) and the success of CRISPR-based clinical applications will largely depend on the further development of suitable carriers for delivering the CRISPR components, often requiring huge consortium efforts and long-term studies.

**Table 1.** CRISPR-Cas delivery methods.

Major categories	Delivery vehicle	Advantages	Limitations
Physical delivery methods	Microinjection	Guaranteed delivery into cell	Difficult to operate
	Electroporation	Delivery to cell population	Generally *in vitro* only
	Hydrodynamic delivery	Low cost	Traumatic to tissues
	Membrane deformation	Suitable for a variety of target cells	High cost
Viral vectors	AAV	Minimal immunogenicity	Low capacity
	AdV	High efficiency delivery	Inflammatory response
	LV	Persistent gene transfer	Prone to gene rearrangement
Nonviral vectors	Nanoparticles	Virus-free, reformable	Require complex design and more researches
	Cationic lipids	Virus-free, simple manipulation	Toxicity
	Exosomes	High biocompatibility, low immunogenicity	Low loading efficiency

## Off-target effects

Genome editing systems may not only cleave DNA at the target site, but also other regions, which is called off-target effects, possibly limiting the application of Cas proteins or leading to harmful effects.^120^ Off-target effects could result in chromosomal rearrangements, which may inadvertently impact some imperfectly matched genomic loci and limit the application of CRISPR-Cas editing systems for therapeutic purposes.^121^ In addition to interfering with the stability of chromosomes, off-target effects can also disrupt the function of essential human genes and cause vital function loss, resulting in various physiological or signaling abnormalities.^122^ Studies demonstrated that CRISPR-Cas tools may be more prone to off-target effects than some of the other conventional gene-editing methods because a Cas protein is a monomer that can fortuitously facilitate recognition of shorter target sequences, while the TALEN and ZFN assemblies are dimeric.^123^ Off-target effects generally result from mismatch recognition by guide RNA and bystander cleavage (not intentionally designed) by Cas enzymes.

A variety of techniques has been tested or used to identify and quantify off-target effects resulted from different CRISPR/Cas genome-editing processes. Within them, whole genome sequencing (WGS) is a direct and unbiased approach of evaluating mutations. However, the sensitivity of WGS cannot meet the requirement for detection of off-target effects in bulk populations, so it offers a useful option to measure the off-target effects with a high frequency in clones and single cells.^124^ In addition, DISCOVER-Seq is an unbiased, sensitive, and powerful method to identify off-target sites in cells and tissues, which has the advantages of low false-positive rates and wider applicability to a range of systems. The *in vivo* application of DISCOVER-Seq after gene editing of adenovirus has been reported, which means it has the possibility for real-time detection of off-targets during the process of gene editing.^125,126^ Other techniques of detecting off-target effects include Bless, Digenome-seq, GUIDE-seq, CIRCLE-seq, SITE-Seq, GOTI, EndoV-seq, etc. The advantages and limitations of these techniques have been described in detail by Manghwar et al.^123^

To counter the possibility of being off-target, several methods have been exploited to design optimum guide RNA sequences with minimal chances of causing off-targeting.^127,128^ In addition, scientists have made advances in engineering Cas enzymes to improve nuclease specificity.^129,130^ Furthermore, a constantly active CRISPR-Cas system in cells may increase the probability of off-target effects and lead to unpredicted sequels. Inhibition of Cas enzymes is demonstrated to be an effective means of reducing off-target effects that has gained intense attention in recent years.^131^ Three inhibitor technologies have been developed based on small-molecule Cas inhibitors,^132^ anti-CRISPR (Acr) proteins,^133^ and small nucleic acid-based CRISPR inhibitors.^134^ Different to the genetic methods by expressing protein-based anti-CRISPRs, small-molecule inhibitors may show fast kinetics and can inhibit the activity of Cas enzymes within a few minutes, which provides the potential of precise temporal control. Small-molecule inhibitors also have the advantages of being cell-permeable, proteolytically stable, reversible, and nonimmunogenic.^135^ However, inexpensive, high-throughput, and sensitive assays are currently unavailable and a huge number of alternative options need to be identified, hence the screening of small inhibitors remains challenging. In particular, design and optimization with medicinal chemistry require substantial time, costs, and specialized knowledge, which may hinder development.^136^ We predict that Acrs may be a highly potent approach as the Acr proteins generally possess several CRISPR-Cas interaction sites and function through a diversity of mechanisms, such as inhibiting the binding of Cas with the target DNA or preventing the enzyme from cleaving the target DNA sequence. This feature of Acrs could make it possible to control the Cas activity and improve the targeting specificity. Nevertheless, the discovery of Acrs is a great challenge as Acrs are extremely heterogeneous and have very few conserved sequences or structures. Like Cas proteins, the delivery of Acr proteins is a challenge because this may add to the burden to cause immune responses by the host.^131^ Recently, scientists have explored newer approaches, such as small nucleic acids as potential inhibitors of CRISPR-Cas systems, which are significantly smaller than natural Acr proteins and easier to deliver *in vivo*. Despite the small size, they have shown the ability to bind the Cas ribonucleoproteins with high affinity, possibly exceeding that of Acr proteins.^137^ For clinical applications, small nucleic acids as Cas inhibitors have some obstacles to overcome, such as degradation by cellular nucleases, serum, renal clearance, and the immune response to any components of their needing to be detected.^138^

As off-target effects are a critical concern for *in vivo* application at the bedside, more applied research is urgently required to help mitigate or avoid these unwanted effects.

## Immunogenicity

*In vivo* delivery of CRISPR systems can induce immune reactions toward the foreign materials through inducing significant innate immunity and/or adaptive immunity (humoral immunity and cellular immunity) in humans.^139^ Innate immune responses might be triggered by guide RNAs. To overcome this issue, phosphatase treatment of *in vitro*–transcribed guide RNAs is designed, which showed subdued innate immune responses without impairing the role of guide RNAs.^140^ Unfortunately, a major concern is that humans may have pre-exposure to the same antigens of Cas nuclease effectors (e.g. Cas9) and/or delivery vectors (e.g. adenoviral vectors) that are needed to carry the effectors for the target treatment.^141,142^ The exposure to these materials may trigger the body's humoral immunity mediated by antibodies or cellular immunity directed by cytotoxic T cells, which may result in severe adverse reactions, death of Cas-expressing cells and treatment failure.^143,144^ Engineering the Cas protein to create a low-immunogenicity Cas effector and developing new delivery vectors with lower chances of prior exposure in humans may be possible strategies to minimize the immune responses, improve safety, and maximize therapeutic effects of the CRISPR-Cas system. Importantly, prior to every clinical trial, one may have to detect related immune reaction components, such as antibodies against Cas proteins and T cells reacting with Cas proteins or vectors, and continue to monitor immune responses during treatment.

## Conclusions

**Figure 4.**
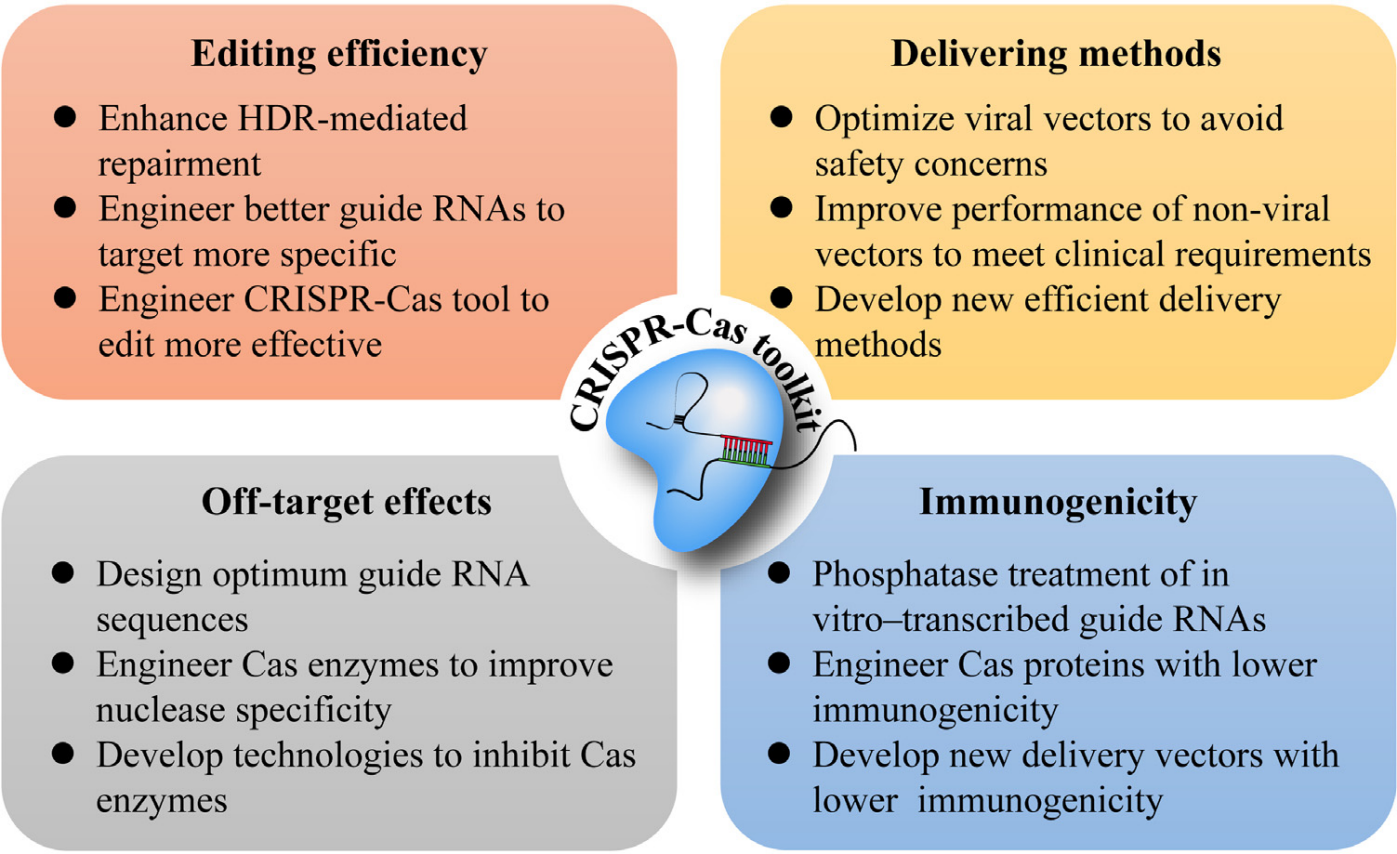
Challenges and possible solutions for CRISPR-Cas precision medicine in clinics. Representative methods or approaches are listed from numerous potential solutions that have being tested.

The ideal gene therapy should be cost-efficient, simple, specific, fast, portable, easy to operate, safe, and highly effective. The CRISPR-Cas systems have quickly blossomed into the most-watched tool for precise gene editing, and they have been recognized among the greatest tools for therapeutic gene editing for diverse diseases. There is still a long road ahead for treatment application for many eagerly awaiting patients with currently incurable diseases, due to the above-mentioned challenges and obstacles (Fig. 4). The ongoing discovery of CRISPR-Cas systems contributes to the expansion of our toolkits. As a novel genome-editing approach, CRISPR-based technologies have already shown some improved outcomes to ameliorate or cure disease. Of course, in order for CRISPR-based toolkits to be used clinically, future studies are needed to develop the ideal approach for efficient delivery, specific expression in relevant tissue, and effective gene correction as well as minimal off-target effects and immunogenicity. In addition, CRISPR gene-editing tools will need to reach the standard of clinical care for diseases with those approved therapeutics and treatments.

In summary, considering the multiple advantages of CRISPR-Cas technology for modifying genomes over other earlier approaches, and the enthusiastic efforts of scientists from all corners of the universe, we may anticipate that the CRISPR-Cas system would eventually realize the great potential to ameliorate or cure a wide range of human diseases in the future.
